# Comparative analysis of sugarcane root transcriptome in response to the plant growth-promoting *Burkholderia anthina* MYSP113

**DOI:** 10.1371/journal.pone.0231206

**Published:** 2020-04-08

**Authors:** Mukesh Kumar Malviya, Chang-Ning Li, Manoj Kumar Solanki, Rajesh Kumar Singh, Reemon Htun, Pratiksha Singh, Krishan K. Verma, Li-Tao Yang, Yang-Rui Li

**Affiliations:** 1 Key Laboratory of Sugarcane Biotechnology and Genetic Improvement (Guangxi), Ministry of Agriculture, Sugarcane Research Center, Chinese Academy of Agricultural Sciences, Guangxi Key Laboratory of Sugarcane Genetic Improvement, Sugarcane Research Institute, Guangxi Academy of Agricultural Sciences, Nanning, Guangxi, China; 2 Guangxi Key Laboratory of Crop Genetic Improvement and Biotechnology, Nanning, Guangxi, China; 3 Department of Food Quality & Safety, Institute for Post-Harvest and Food Sciences, The Volcani Center, Agricultural Research Organization, Rishon LeZion, Israel; 4 Department of Biotechnology, Mandalay Technological University, Mandalay, Myanmar; 5 College of Agriculture, Guangxi University, Nanning, Guangxi, China; ICAR-Indian Institute of Agricultural Biotechnology, INDIA

## Abstract

The diazotrophic *Burkholderia anthina* MYSP113 is a vital plant growth-promoting bacteria and sugarcane root association. The present study based on a detailed analysis of sugarcane root transcriptome by using the HiSeq-Illumina platform in response to the strain MYSP113. The bacterium was initially isolated from the rhizosphere of sugarcane. To better understand biological, cellular, and molecular mechanisms, a de novo transcriptomic assembly of sugarcane root was performed. HiSeq-Illumina platformwas employed for the sequencing of an overall of 16 libraries at a 2×100 bp configuration. Differentially expressed genes (DEGs) analysis identified altered gene expression in 370 genes (total of 199 up-regulated genes and 171 down-regulated genes). Deciphering the huge datasets, concerning the functioning and production of biological systems, a high throughput genome sequencing analysis was attempted with Gene ontology functional analyses and the Kyoto Encyclopedia of Genes and Genomes (KEGG) pathway analysis. The report revealed a total of 148930 unigenes. 70414 (47.28%) of them were annotated successfully to Gene Ontology (GO) terms. 774 at 45 days, 4985 of 30 days and 15 days of 6846 terms were significantly regulated. GO analysis revealed that many genes involved in the metabolic, oxidation-reduction process and biological regulatory processes in response to strain MYSP113 and significantly enriched as compare to the control. Moreover, KEGG enriched results show that differentially expressed genes were classified into different pathway categories involved in various processes, such as nitrogen metabolism, plant hormone signal transduction, etc. The sample correlation analyses could help examine the similarity at the gene expression level. The reliability of the observed differential gene expression patterns was validated with quantitative real-time PCR (qRT-PCR). Additionally, plant enzymes activities such as peroxidase and superoxide dismutase were significantly increased in plant roots after the inoculation of strain MYSP113. The results of the research may help in understanding the plant growth-promoting rhizobacteria and plant interaction.

## Introduction

Sugarcane crop of the family Poaceae globally fulfills the need for sugar, bioethanol, animal feed, and molasses [1–3]. Sugarcane cultivation requires a large amount of nitrogen (N) fertilizers that varies nearly 120 to 320 kg ha^-1^ or more. Though synthetic nitrogen is employed in the cultivation of sugarcane, only a small amount of nitrogen is taken by sugarcane, and the remaining may lead to soil and water pollution. Microbes through biological nitrogen fixation can overcome the problem of synthetic nitrogen [4]. Diazotrophic microbes that can grow under nitrogen limiting condition and fix the gaseous nitrogen to available for a plant that could be the best alternative for this problem [5, 6]. The microbes that are located in the rhizosphere are termed rhizobacteria or plant growth-promoting rhizobacteria (PGPR). There is a specific relationship between rhizosphere bacteria and sugarcane.

*Burkholderia* genus abundantly proliferates in a broad range of ecological niches and well-known plant-associated bacteria. Several species of Burkholderia were reported as PGPR in different plants viz., tomato [7], amaranthus [8], maize [9], rice [10], and sugarcane [11]. PGPR promote sugarcane growth through different mechanisms such as nitrogen fixation, production of plant hormone, antibiotic production, etc. [12]. PGPR are capable of producing antibiotics against plant pathogens and promote plant growth by imparting resistance to pathogens [13, 14]. PGPR produces enzymes, secondary metabolites, phytohormones and helps in the growth and development of roots of the plant. It also controls root physiology by altering gene transcription and metabolite biosynthesis in cells of the plant. Alterations in the architecture of root may be produced by the interference of PGPR with major hormonal pathways (auxin, ethylene, cytokinin, gibberellin, and abscisic acid) which are involved in the development of plant root [15–18]. However, the exact mechanism involved between the plant and PGPR interactions remains unclear [19]. The plant and PGPR relationships are complex, and this relationship varies depending on the soil-inhabiting microbial population and plant metabolism. Next-Generation Sequencing (NGS) has enabled understanding the taxonomic and functional behavior or diversity of PGPR groups. A probable development of meta transcriptomics and meta proteomics could shed knowledge on the ecological functioning of PGPR within the rhizosphere [20]. Recent technological developments have the potential to increase significantly the knowledge of sugarcane plants through the application of emerging genomic technologies, and the use of NGS could have significant implications for crop genetics and breeding. Transcriptome sequencing can provide information regarding the gene content of a species and can complement genome sequencing approaches [21]. RNA sequencing (RNA-Seq) has been applied as a tool for transcriptome analysis in many species, such as *Arabidopsis thaliana*, [22], *Brassica* spp. [23], rice [24], maize [25], and sugarcane [21]. Recently PGPR-responsive transcriptome analyses have been done in different crops under salt stress, help to understand the molecular basis of plant-PGPR interaction [26, 27]. Therefore, more studies needed to unlock the complex interactions of plant-PGPR. Also, studies focusing on the part of *Burkholderia anthina* have not been reported so far. Our previous work reported that this bacterium has an ability to produce siderophore, solubilize phosphate, secret Indole acetic acid (IAA), and produce ACC deaminase activity as well as able to colonize in the plant root, stem, and leaf, and increase the growth and biomass of sugarcane plants [11]. In a first-of-its-kind attempt, the current research focuses on the comparative transcriptome analysis of sugarcane root in response to *Burkholderia anthina* MYSP113 to identify its association and to elucidate its role in promoting sugarcane growth.

## Materials and methods

### Plant material and bacterial inoculation

**Fig 1:**
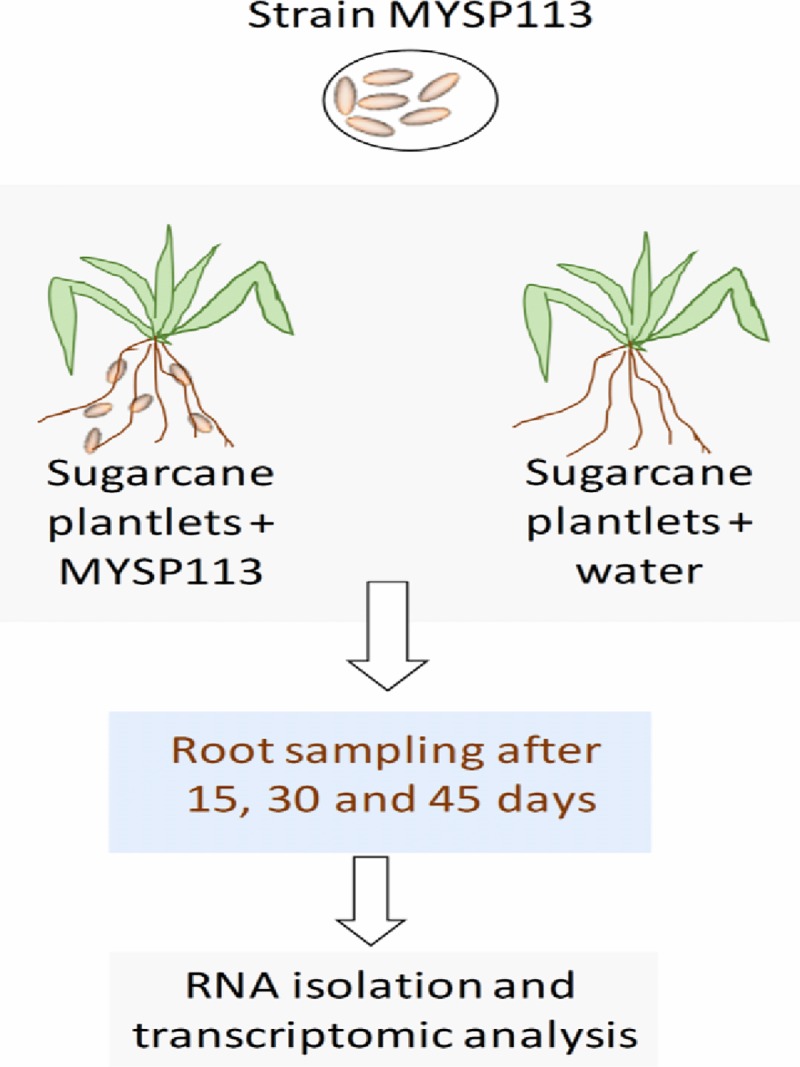
Schematic presentation of the RNA isolation and transcriptome analysis of sugarcane plant roots.

Micro-propagated sugarcane plantlets were developed from the stem apical meristems of China commercial sugarcane cultivar GXB-9, according to Wang et al. [28]. Bacterial strain MYSP113 was previously isolated from the sugarcane rhizosphere and characterized by Malviya et al. [11]. Inoculation and acclimatization of micro-propagated plantlets were performed according to Lin et al. [29]. Strain MYSP11 was grown overnight in Luria Britani broth and cells were harvested by centrifugation. Rooted plantlets were co-cultured with bacteria of 2.0×10^6^ cells per milliliter in liquid 1/10 Murashige and Skoog (MS) medium; plantlets without inoculation were prepared as the control. After two days of inoculation, the plantlets were transferred into the autoclaved sand and perlite mixtures (1:1, v /v), and acclimatized in a growth cabinet (28°C, 16/8 h day/night, 400 μ mol m^−2^ s^−1^) for 7 days. Then the similar sizes plantlets were selected and transplanted into pots containing 3.5 kg of field soil with moderate fertility (each kg soil containing 20.31 g organic matter, 135 mg available N, 86 mg available P, 79 mg available K,0.86 total N, 3.12 g total P, 7.23 g total K and pH was 6.17). The plants were grown in a greenhouse with the temperature maintained between 22 to 30°C and the relative humidity between 50 to 80%. As per the requirement, sterile water was used for the irrigation of sugarcane plants. The root samples with bacterial inoculum were collected at 15 days, 30 days, and 45 days (IT15, IT30, and IT45) after transplant. Likewise, the control roots were also collected at 15 days, 30 days, and 45 days (CT15, CT30, and CT45). A schematic diagram represents the overall experiment design of transcriptome analysis of sugarcane root after inoculation of *Burkholderia*strain (Fig 1).

### RNA isolation

Total RNA was isolated from the root using the commercial TRIZOL (Invitrogen, Carlsbad, CA, USA) reagent according to the instructions of the manufacturer's protocol. RNA samples were treated with diethyl pyrocarbonate-treated H_2_O. The quality of RNA was assessed on 1% agarose gels. Before the submission for sequencing, the quality and quantity of RNA were verified using Nano Photometer® spectrophotometer (IMPLEN, CA, USA) and RNA Nano 6000 Assay Kit of the Bioanalyzer 2010 system (Agilent Technologies, CA, USA). The concentration of RNA was measured using the Qubit® RNA Assay Kit in Qubit® 2.0 Fluorometer (Life Technologies, CA, USA).

### Library preparation

RNA-Seq library construction, RNA-Seq, and bioinformatics analysis were performed as described previously [30]. An amount of 1.5 μg of total RNA was used for sample preparation. The samples were subjected to library preparation using NEBNext® Ultra^™^ RNA Library Prep Kit for Illumina® (NEB, USA) following the manufacturer’s instructions and index codes were added for each sample. The experimental process was continued by purifying mRNA from 1.5 μg of total RNA using poly-T oligo-attached magnetic beads and then the fragmentation process was carried out using divalent cations under elevated temperature in NEB Next First Strand Synthesis Reaction Buffer (5X). After fragmentation, the first and second cDNA strands were synthesized. The first strand of cDNA was synthesized using random hexamer primer and MMuLV and MMuLV Reverse Transcriptase (RNase H-). The second strand was synthesized using DNA Polymerase I and RNase H by using exonuclease/polymerase activities, and the overhangs were converted into blunt ends. After the adenylation of 3' end of DNA fragments, ligation with NEBNext Adaptor with a hairpin loop structure was conducted for the preparation of hybridization. The library fragments were purified with AMPure XP system (Beckman Coulter, Beverly, USA) to select 150–200 bp in length cDNA fragments. Before performing PCR amplification, the adaptor-ligated cDNA at 37°C for 15 min, followed by 5 min at 95°C was subjected to 3μl USER Enzyme (NEB, USA). The procedure was followed by PCR amplification conducted with Phusion High-Fidelity DNA polymerase, Universal primers and Index(X) Primer. The PCR products were purified and quality was assessed by the AMPure XP system and Agilent Bioanalyzer 2100 system.

### Cluster generation and sequencing

These libraries were subjected to the generation of clusters through cBot Cluster Generation System using TruSeq PE Cluster Kit v3-cBot-HS (Illumia) according to the manufacturer’s protocol. After cluster generation, Hiseq sequencing was performed on the Illumina platform. The raw data was generated after image analysis, base calling, and quality check [31]. The qualified raw data have been deposited at Sequence Read Archive (SRA) (http://www.ncbi.nlm.nih.gov/sra/) under accession number PRJNA606520.

### *De novo* transcriptome assembly and differential expression analysis

Transcriptome assembly was accomplished based on the left.fq and right.fq using Trinity [32] with min_kmer_cov set to 2 by default and all other parameters set default. The assembled transcripts were hierarchically clustered to unigenes using shared reads and expressions by Corset [33]. Differential expression analysis was performed using the DESeq R package (1.18.0). DESeq provides statistical routines for determining differential expression in digital gene expression data using a model based on the negative binomial distribution.

### Gene Ontology and KEGG enrichment analysis of differentially expressed genes

Gene Ontology (GO) enrichment analysis of differentially expressed genes was implemented by the GOseq Rpackage, in which gene length bias was corrected. GO terms with a corrected p-value less than 0.05 were considered significantly enriched by differential expressed genes [34]. KEGG is a database resource for understanding high-level functions and utilities of the biological system, such as the cell, the organism, and the ecosystem, from molecular-level information, especially large-scale molecular datasets generated by genome sequencing and other high-throughput experimental technologies (http://www.genome.jp/kegg/). We used KOBAS software to test the statistical enrichment of differential expression genes in KEGG pathways.

### Antioxidant enzyme activity determination

Antioxidant enzymes activity of sugarcane root was measured at 15, 30, and 45 days after inoculation of strain. Antioxidant enzymes activity of sugarcane root were measured according to Xu et al. [35]. After treatment, the roots excised from the seedlings were immediately frozen in liquid nitrogen and were finely ground into a powder with a pestle. Then, 100 mM phosphate buffer (pH 7.0) was added, and the final concentration of the samples was 10% (w/v). The samples were then vortexes and centrifuged at 3500 g for 10 min at 4°C. The supernatants were collected and the protein content was analyzed with a bicinchoninic acid (BCA) kit. The activities of superoxide dismutase (SOD), peroxidase (POD), catalase (CAT) and polyphenol oxidase (PPO) were determined according to the protocols of the assay kit respectively, obtained from Bioengineering Suzhou Keming Biotechnology Co., Ltd., China.

### Gene validation by qRT-PCR

Total RNA of different root samples was extracted by the Plant RNA Extract Kit (TIANGEN, Beijing, China), and approximately 0.5 μg of total RNA was used for cDNA synthesis using Prime Script RT reagent kit (Takara, Dalian, China). Gene-specific primers for qRT-PCR are listed in S1 Table and were synthesized by Sangon Biotech (Shanghai, China). qRT-PCR was performed on an ABI 7500 real-time PCR system (Applied Biosystems, Forster City, CA, USA) using SYBR premix Ex Taq (Takara, Japan). The sugarcane glyceraldehyde-3-phosphate dehydrogenase (GAPDH) gene, as an internal control, was used to normalize the expression levels of the target genes [36]. Three technical replicates were carried out for each sample, and the mean expression level and standard deviation were calculated using the 2^−ΔΔCt^ method [37].

### Data analysis

In the case of samples with biological replicates, differential expression analysis of two conditions/groups (two biological replicates per condition) was performed using the DESeq R package (1.18.0) [38]. DESeq provides statistical routines for determining differential expression in digital gene expression data using a model based on the negative binomial distribution [39]. The resulting P-values were adjusted using the Benjamini and Hochberg’s approach for controlling the false discovery rate. Genes with an adjusted P-value <0.05 found by DESeq were assigned as differentially expressed. On the other hand, for samples without biological replicates, prior to differential gene expression analysis, for each sequenced library, the read counts were adjusted by edgeR program package through one scaling normalized factor.

## Results

**Fig 2:**
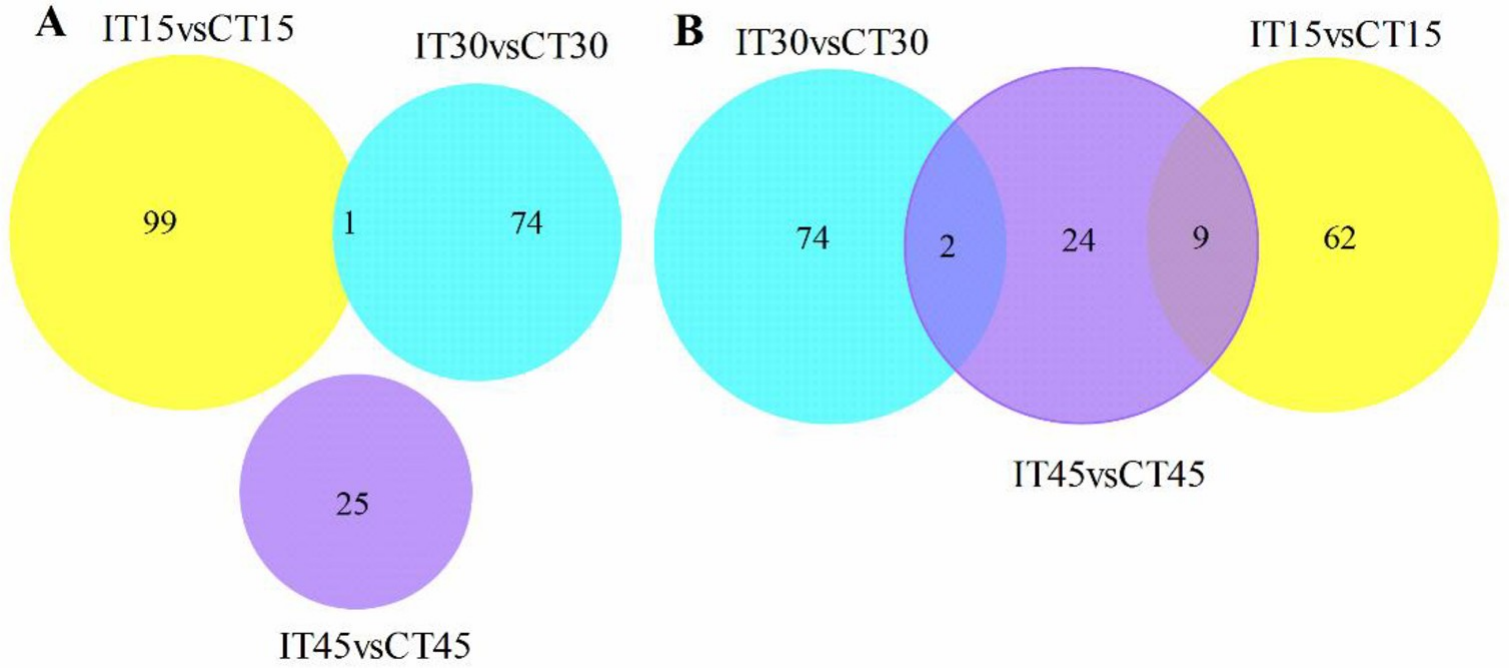
Venn diagram of differentially expressed gene sets across three distinct groups. (A) represents up-regulated genes (B) represents down-regulated genes.

A total of 144.28 Gb data was obtained from the 36 libraries, and after assembly, we total get 314967 unigenes, and the genes’ annotation results in seven different databases present in S2 Table. Raw data (raw reads) of fastq format were firstly processed through in-house perl scripts. In this step, clean data (clean reads) were obtained by removing reads containing adapter, reads containing ploy-N and low quality reads from raw data. At the same time, Q20, Q30 and GC content the clean data were calculated. All the downstream analyses were based on clean data with high quality. The original raw data from Illumina HiSeqTM are transformed into Sequenced Reads by base calling. Raw data are recorded in a FASTQ file, which contains sequence information (reads) and corresponding sequencing quality information. Differential gene expression analysis identified altered gene expression in 370 genes (total of 199 up-regulated genes and 171 down-regulated genes) (Fig 2) in 3 distinct groups of sugarcane root samples inoculated with strain MYSP113. Venn diagram in Fig 2A represents a total of 199 genes that were up-regulated in three groups, and only one gene was observed in common between two groups (IT15 vs CT15 and IT30 vs CT30). A total of 171 genes were seen in the down-regulated gene set (Fig 2B) across the three groups. Eleven (6.4% of down-regulated gene data) genes were found common in the three groups which may be assumed to be associated with transcriptional changes. Fig 2 suggests a significant proportion of genes exhibited altered gene expression upon interaction with the strain MYSP113.

### Filtering the differential gene expression

**Fig 3:**
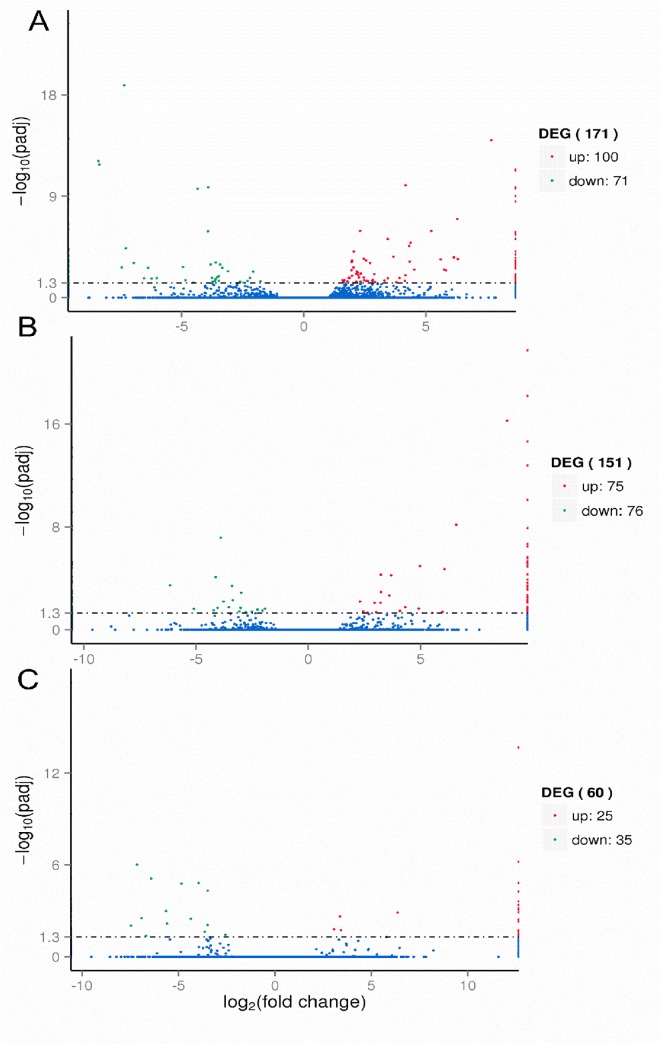
Volcano plots of Differentially Expressed gene sets across three distinct groups (A) IT15vsCT15 (B) IT30 vsCT30 (C) IT45vsCT45. The x-axis shows the fold change in gene expression between different samples, and the y-axis shows the statistical significance of the differences. Statistically significant differences are represented by red dots.

Volcano plots are used to infer the overall distribution of differentially expressed genes (Fig 3). For experiments with biological replicates, as the DESeq already eliminates the biological variation, our threshold is normally set as p < 0.05.cA total of 171 genes were expressed in the gene set, 100 were up-regulated and 70 were down-regulated in group1 (Fig 3A), while 151 genes were regulated in group 2 in which 75 and 76 genes were up and down-regulated, respectively (Fig 3B). In group 3 only 60 genes were expressed in which 25 were up-regulated and 35 down-regulated (Fig 3C).

### Cluster analysis of differentially expressed genes (DEGs)

**Fig 4:**
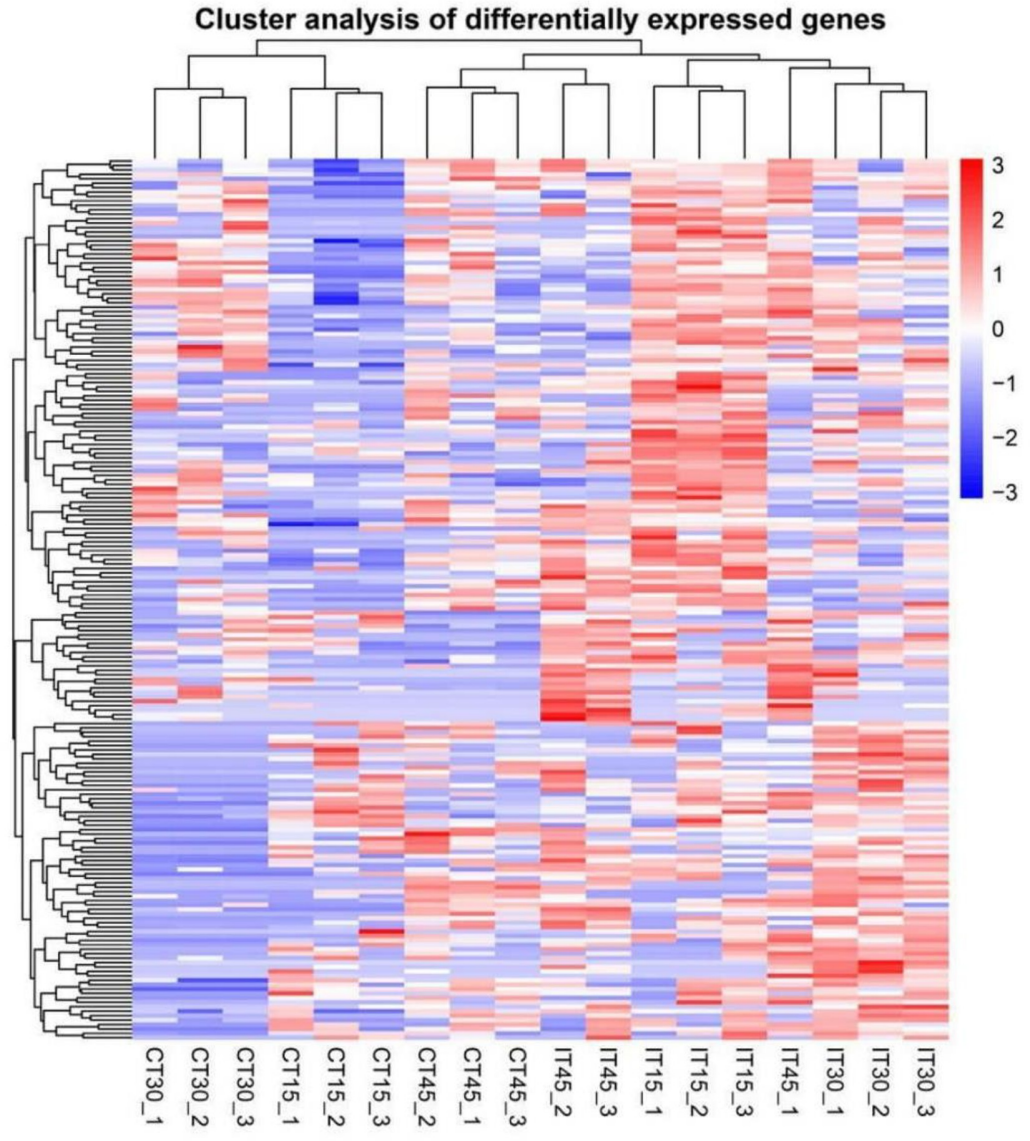
Heatmap is representing differential expression between samples. Red represents genes with high expression values, and blue represents genes with low expression values. Each treatment was replicated three times and exhibiting similar expression in each.

After identification of sugarcane DEGs analysis of gene expression levels, the DEGs were quantified by DESeq R package software v1.20.0, all read counts were normalized to FPKM (Fragments Per Kilobase of transcript sequence per Millions of base pairs sequenced) [40]. Dynamic changes of gene expression patterns were recovered by using a gathering cut-off value of fold change (FC) *>*2 and an adjusted p-value (FDR) of *<*0.05. By similar expression kinetics, 370 DEGs were depicted by a heatmap analysis to show significant clusters of DEGs according to tissue specificity and time treatment (Fig 4). Similar expression profiles were observed for replicated samples, and specific clusters were denoted for examples (groups 1, 2 & 3) depending on sugarcane root responses.

### GO classification

**Fig 5:**
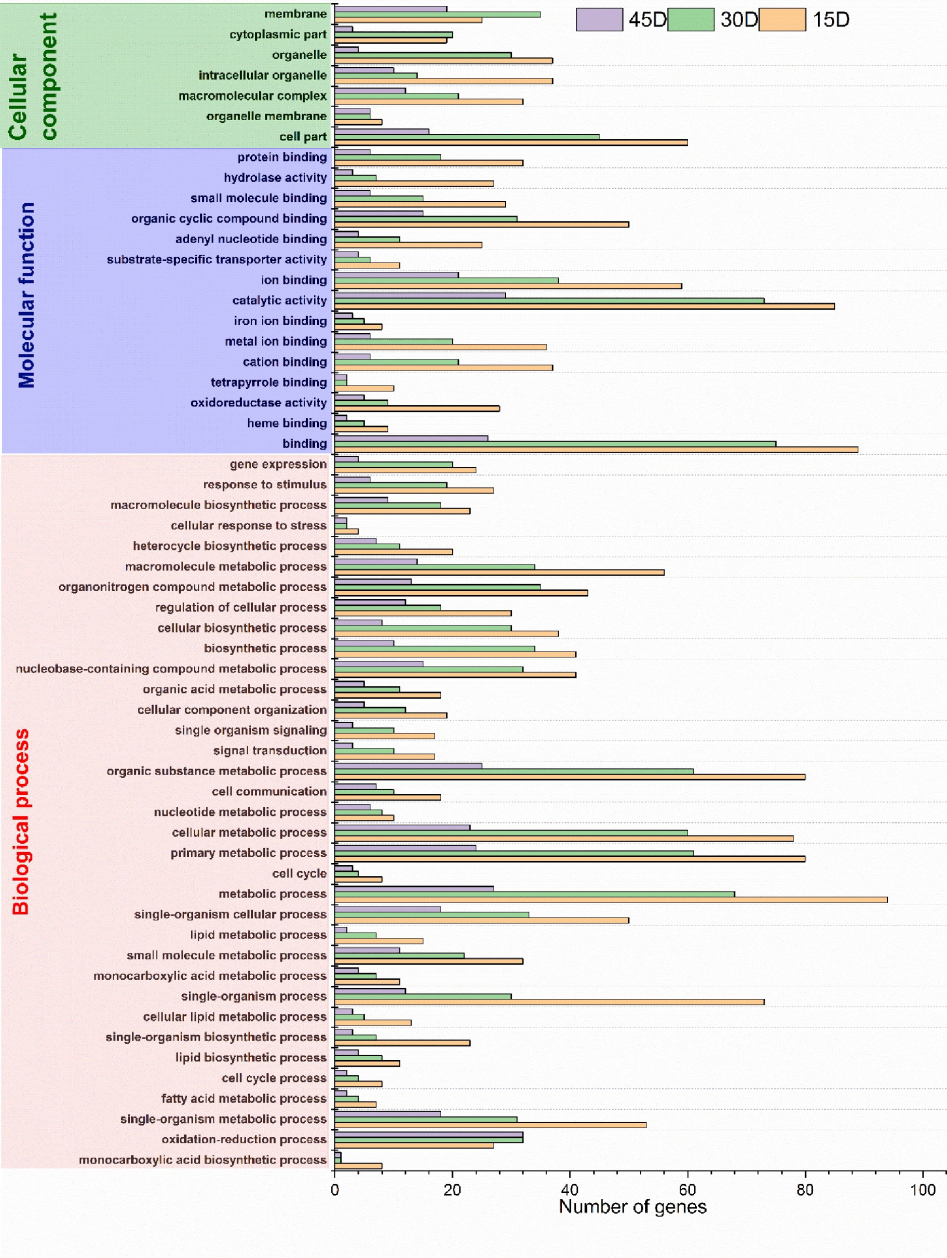
Gene Ontology (Go) classification of a differentially expressed genes. The Go term was categorized into biological process, cellular component, and molecular function. 15D, 30D and 45D indicated differentially expressed genes at days 15, 30 and 45 sugarcane roots in the response of strain MYSP113 inoculation.

Gene Ontology (GO) is an international standardized gene function classification system that describes the properties of genes and their products in any organism. To analyze the sugarcane enriched functional GO terms in response to strain MYSP113, the BLAST2 GO tool was used [41]. The analysis revealed a total of 148930 unigenes. 70414 (47.28%) of them were annotated successfully to GO terms. 774 of IT45vsCT45, 4985 of IT30vsCT30 and IT15vsCT15 of 6846 terms were significantly regulated. All transcripts were then assigned into three main GO categories: biological process, cellular component, and molecular function. Comparing the up-regulated and down-regulated groups, it was observed that some of the genes had more significant enriched GO terms than the control. At 15 days, single organism process and carbohydrate derivative metabolism in biological process and oxidoreductase activity in molecular function was most enriched in the down-regulated DEGs, while in the up-regulated DEGs, metal ion and cation binding in molecular function and catalytic complex in cellular component were most enriched. At 30 days, pathogenesis and development process in the biological process were most enriched in the down-regulated DEGs while the alpha-amino acid catabolic process in biological process and glucosyltransferase activity in molecular function were most enriched in the up-regulated DEGs. At 45 days, an oxidation-reduction process in the biological process was most enriched in the down-regulated DEGs while cellular carbohydrate process in biological process, the primary active trans-membranein molecular function was most enriched in the up-regulated genes (S1–S3 Figs). Go enrichment results at 15, 30, and 45 days showed that some of the genes were significantly improved compared to the control. The catalytic activity and binding terms (in molecular function category), cell part (in cellular component) and signal organism process and metabolic process terms (in biological process category) were significantly enriched in GO categories (Fig 5). The differentially expressed genes were primarily involved in catalytic activity, molecule activity, transporter activity, binding, antioxidant activity, enzyme regulator activity, transcription regulator activity, signal transduction, and molecular transducer activity.

### KEGG pathway analysis

**Fig 6:**
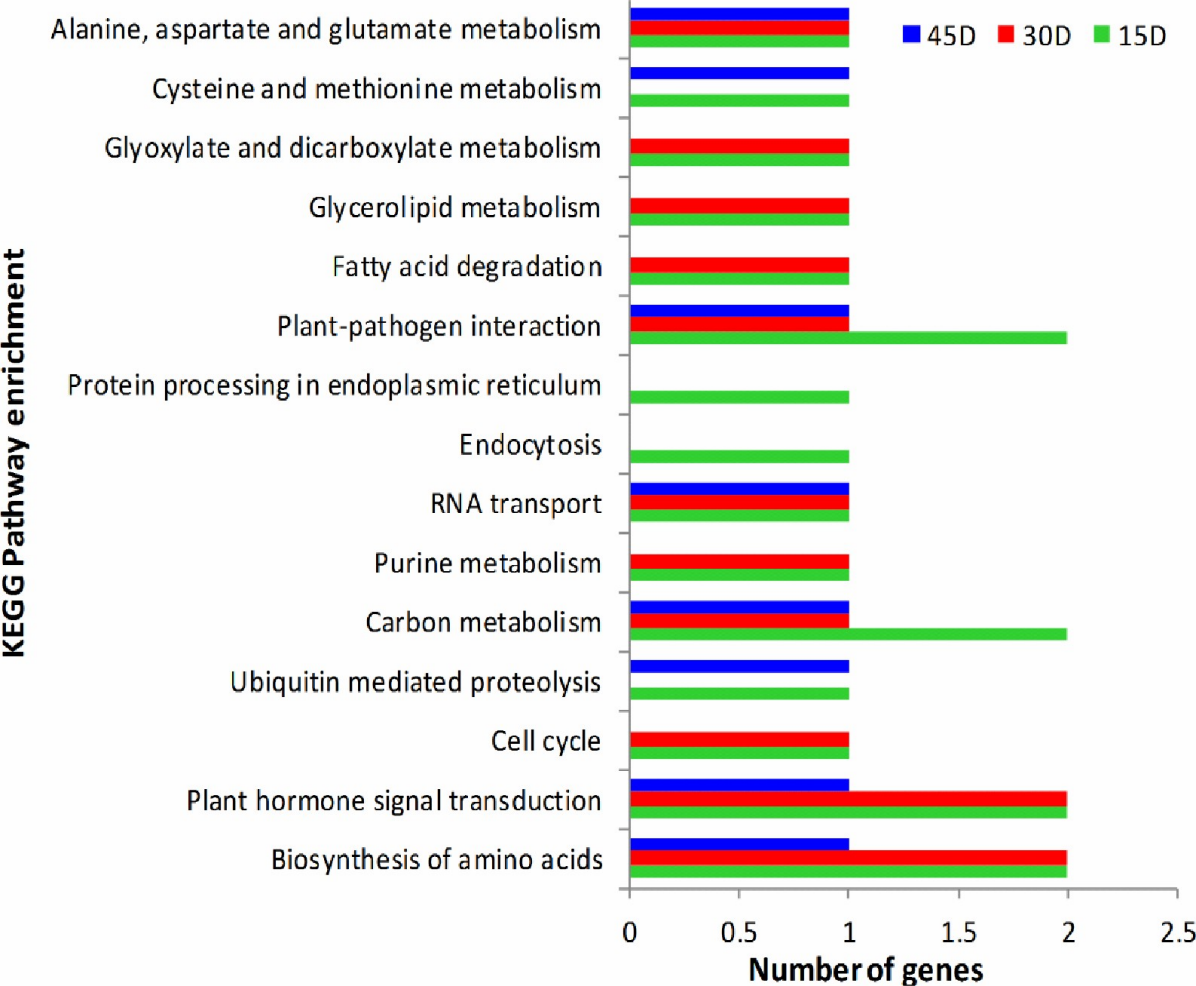
Prime pathway of KEGG enrichment analysis. 15D, 30D and 45D indicated differentially expressed genes at days 15, 30 and 45 sugarcane roots in the response of strain MYSP.

The mapping of metabolic pathways provided by the KEGG provides valuable classifications usable in interpreting the complex biological functions of genes [42]. Using the KOBAS2.0 software, a total of 1449 genes annotated were associated with 20 predicted KEGG metabolic pathways [31]. In summation, the DEGs were significantly enriched in 20 KEGG metabolic pathways, using the criteria of P-values*<*0.05. Among them, eight pathways for IT15vsCT15 group, five pathways for IT30vsCT30 group and one pathway for IT45vsCT45 group were improved considerably in the up-regulated DEGs. In the down-regulated DEG set, six pathways for IT15vsCT15 group, 17 pathways for IT30vsCT30 group, six pathways for IT45vsCT45 group were significantly enriched (S4–S6 Figs). The RNA degradation pathway was enriched considerably in both the up-regulated and down-regulated DEGs. The prime pathways with the most representative of genes were presented in Fig 6. The KEGG enrichment analysis results showed that the some of the metabolic pathways, are involved in the biosynthesis of amino acid, plant hormone signal transduction, plant-pathogen interaction, alanine, carbon metabolism, fatty acid degradation, and aspartate and glutamate metabolism, were significantly regulated in sugarcane in response to strain MYSP113 exposure.

### Correlation between samples

**Fig 7:**
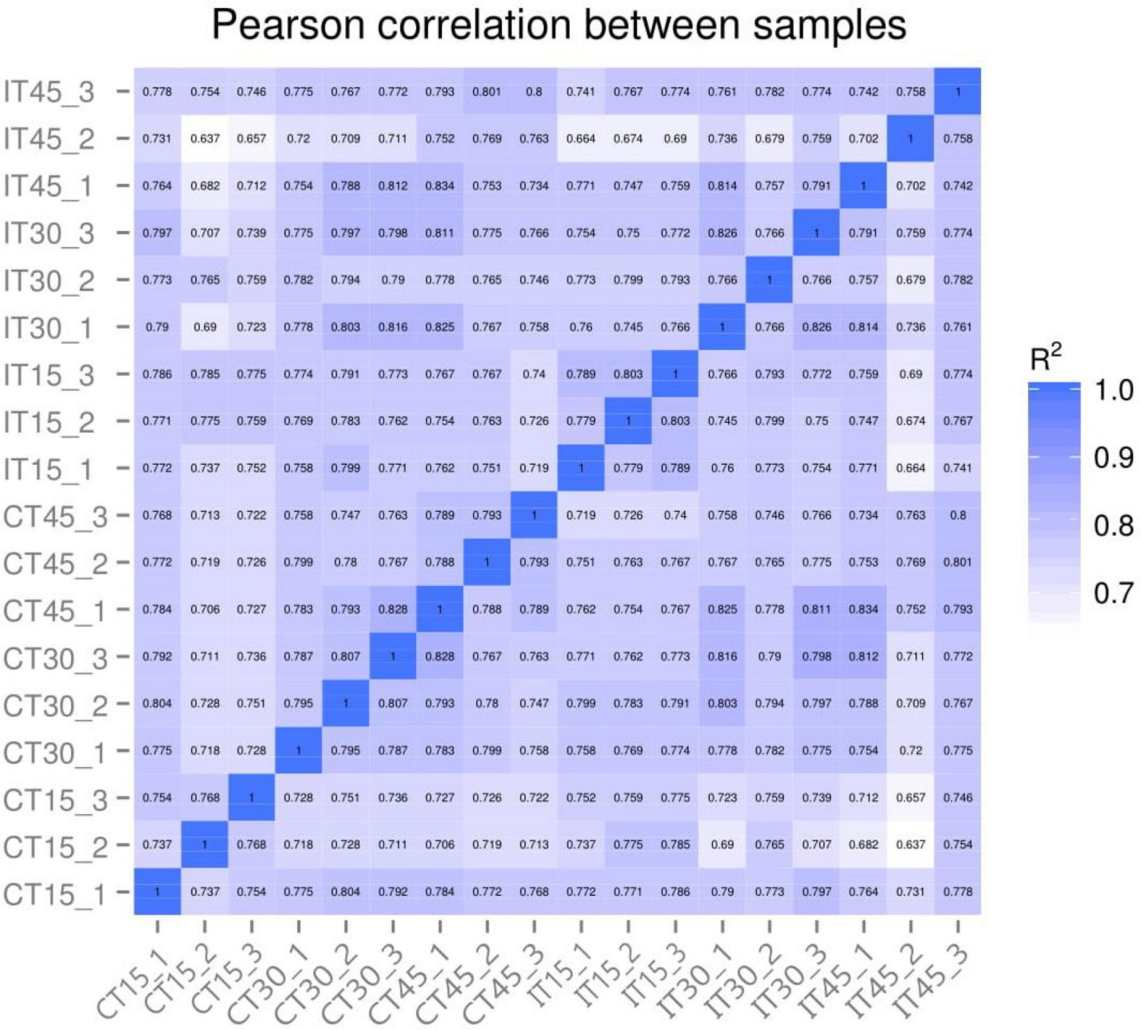
Pearson correlation between samples.

A Pearson's correlation coefficient analysis was employed to compare Log2 of RPKM in repose relative to Log2 of RPKM in rep 2 in both control and inoculated plants while aiming at analyzing the expression level of transcripts and the pairwise comparisons between biological replicates. These computational results showed an R2 = 1.0 (Fig 7) correlation between the two replicates. These correspond to a great confident relationship, indicating that the biological replicas have good reproducibility.

### *De novo* transcriptome assembly and differential expression analysis

Using Trinity, 540,736 *de novo* assembled transcripts were obtained with an average length of 782 bp and an N50 value of 1388 bp (Table 1). The estimated DNA size of bacterium~7.3 Mb and the assembly generated in this study covered 4.23%. Additionally, these transcripts were clustered into 314,967 unigenes with a mean length of 1147 bp and an N50 value of 1661 bp (Table 2). Among the 87,437 unigenes, the two most effective sizes were those that ranged from 200 to 500 bp (87,437 unigenes or 27.76%) and those that varied in length from 500 to 1000 bp (98,409 unigenes or 31.24%). This was followed by 80,220 unigenes (25.47%) that range from 1000 to 2000 bp and finally by 48,901 unigenes (15.53%) that were more than 2000 bp in length. The sizeable proportion of short unigenes (69.45%) could be attributed to the lack of an available reference genome sequence for Strain MYSP113 [43]. Moreover, the abundance of these short unigenes could be the result of fragmented transcripts or insufficient sequencing depth.

**Table 1:** Summary of transcripts.

Transcripts length	Total number	Percentage
200–500 bp	308982	57.14%
500-1k bp	102549	18.96%
1k-2k bp	80304	14.85%
>2k bp	48901	9%
total number	540736	-
Total length (bp)	422977779	-
N50 length (bp)	1388	-
N90 length (bp)	275	-
Median length (bp)	404	-
Mean length (bp)	782	-

**Table 2:** Summary of unigenes.

Unigene length	Total number	Percentage
200–500 bp	87437	27.76%
500-1k bp	98409	31.24%
1k-2k bp	80220	25.47%
>2k bp	48901	15.53%
total number	314967	-
Total length (bp)	361148697	-
N50 length (bp)	1661	-
N90 length (bp)	536	-
Median length (bp)	813	-
Mean length (bp)	1147	-

### Antioxidant enzyme activity

**Fig 8:**
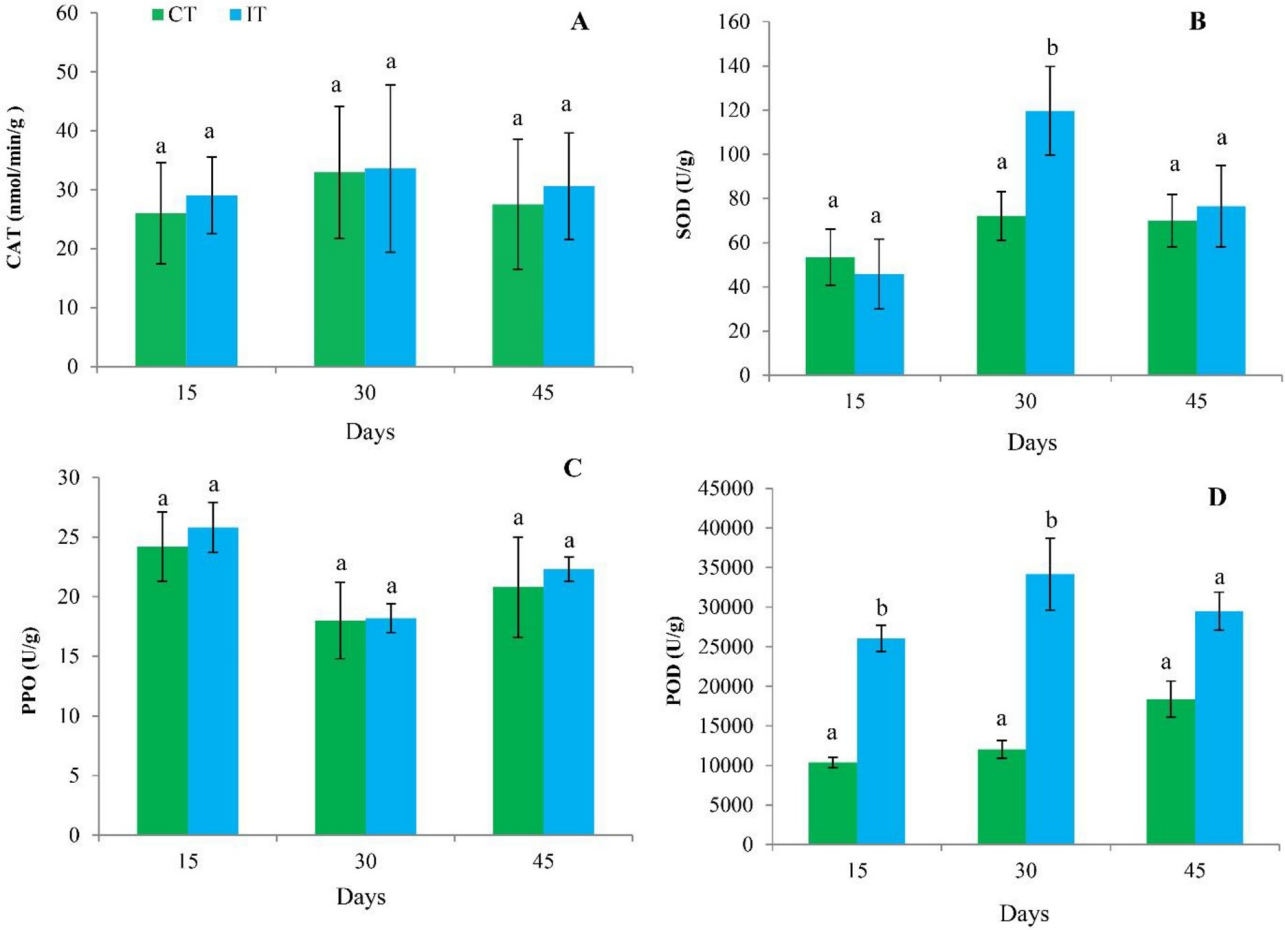
Effect of bacterial inoculation on sugarcane antioxidant defense-related enzymes (A) Catalase (CAT) (B) superoxide dismutase (SOD) (C) Polyphenol oxidase (PPO) and (D) Peroxidase (POD) at 15, 30 and 45 days. Different letters indicate significant differences in the control and bacterial inoculated plants at p ≤ 0.05 according to Duncan’s multiple range test (DMRT) (mean ± SD, n = 5).

We observed that bacterial inoculation increased the activity of antioxidant enzymes (Fig 8). CAT and PPO were not increased significantly but SOD activity was significantly increased at 30 days while POD activity was significantly increased at 15 and 30 days over the control plant roots.

### Gene validation by qRT-PCR

To validate the accuracy and reproducibility of the RNA-Seq results, we randomly selected 15DEGs for qRT-PCR assays, including 7 up-regulated and 8 down-regulated genes in the unigene dataset. The expression levels of selected genes in the qRT-PCR analysis are summarized in S7 Fig. These results were consistent with the data obtained from the RNA-Seq analysis, indicating the reliability of the RNA-Seq.

## Discussion

The current study is a pioneer attempt at comparative transcriptome analysis of sugarcane root in response to strain MYSP113. The study laid a foundation for the understanding of differential gene expression analysis of sugarcane root. To enable the comprehensive characterization of ‘static’ bacterial transcriptomes, it is necessary to generate a pool of different transcripts to obtain the expression of as many genes as possible. This was achieved mainly by the cultivation of strain MYSP113 under three (IT15vs CT15, IT30vs CT30, IT45vs CT45) distinct conditions (at 15, 30 and 45 days after inoculation of strain) and pooling RNA samples before sequencing. 53.78% (199 up-regulated genes among 370 genes) genes were differentially expressed under these conditions. Totals of 314,967 unigenes were assembled and 232348 unigenes were annotated. The observation may also reflect the fact that RNA was combined from cells cultivated under various growth conditions that were chosen with a similar rationale for the strain MYSP113 and may indicate that the same set of genes was transcribed under the preferred terms.

The GO enrichment analysis showed that several of the down-regulated sequences were grouped into many stress-related pathways as a response to the oxidation-reduction process and catalytic process among others. On the other hand, several up-regulated sequences were grouped into in biosynthesis of amino acid. Furthermore, some essential carbohydrate-related pathways were enriched such as metabolic starch process, sucrose metabolic process, cellulose biosynthetic process, and amino acid catabolic process, among others. The GO term cation binding from the molecular function category was the most enriched one in the up-regulated set, while in the down-regulated set the GO term single-organism process, pathogenesis, and oxidation-reduction process in biological process category was enriched significantly. These genes code for peroxidases and catalases, suggesting that these proteins play critical roles during strain MYSP113 invasion into sugarcane. Interestingly, all GO terms in biological process in the down-regulated set were involved in metabolism, indicating that these pathways were strongly affected by the pathogen in sugarcane. Genes involved in the oxidation process are related to stress and would help to eliminate the excessive relative oxygen species (ROS) and maintain the balance of ROS in sugarcane, which might be a mechanism by which sugarcane can cope with stress [44]. It was also identified that most enriched GO terms in molecular function category in the down-regulated set include binding and catalytic processes, suggesting that the plant reduced the energy spent in transcriptional and translational activities in order to conserve the energy demands and focused on other processes involved in defense response [45].

KEGG enrichment analysis revealed that a large number of genes were involved in the pathways related to phenylpropanoid biosynthesis, starch and sucrose metabolism, amino sugar and nucleotide sugar metabolism, and hormone signaling and transduction. These pathways are principally involved in cell wall biosynthesis, cell proliferation, nutrient accumulation, primary metabolism and hormone signaling [46]. The KEGG enrichment analysis also showed that the metabolic pathways and biosynthesis of amino acids were significantly regulated in response to strain MYSP113. The previous study reported that amino acids not only participate as precursors in the synthesis of proteins, but also have critical roles for plants in growth, development, reproduction, defense, and environmental responses [47]. These pathways are of great interest since the most economically important characteristic of sugarcane for biomass production and accumulation of sucrose [48].

In this study, the 5′ UTR length of strain MYSP113 transcripts was shown to be equal or longer than ten nucleotides in 99.75% of the cases, and it peaked at around 30 base pairs. NGS technology enabled the generation of sufficient sequence information within a short period [32]. The *de novo* assembly and characterization of the root transcriptome of sugarcane using Illumina Hisequencing has been reported [49]. The current investigation attempted the transcriptome reconstruction and *de novo* assembly to unveil the enormous amount of novel genomic information of sugarcane root. A total of 540736 good quality transcripts (length ≥ 200, FPKM >1) were obtained with the average transcript length being 782800 bp. The *de novo* assembly produced 314967 unigenes with an average length of 1147 bp. These results were similar to previous work done on sugarcane by [50]. Previous studies have been showing that transcription factors (TFs), protein kinases (PKs), and transporters (TRs) play critical roles in signal transduction pathways involved in important biological processes including plant-microbe interactions [51]. The initial results on the overall transcriptome of sugarcane root could be further substantiated with comparative transcriptome studies about biotic or abiotic stress conditions. Hence, the data generated from this study would be a backbone for functional genomics studies in sugarcane root including, but not limited to, the isolation and characterization of enzymes involved in specific metabolic pathways especially the single-organism metabolic process.

In the present study, antioxidant enzyme activities of sugarcane plant roots were increased as compared to the control. POD and SOD activity was significantly increased in the response of strain MYSP113 inoculation. Increase activity of SOD has been indicating that an increase in the concentration of superoxide radicals because PGPR inoculation can promote the de-novo synthesis of enzymes [52]. PODs are oxidoreductive enzymes that participate in various physiological processes such as auxin catabolism, senescence, suberization, lignifications, linking of cell wall structural proteins, and defense gene activation against pathogens [53]. Plant growth-promoting bacteria are free-living in soil, rhizosphere and beneficial to plants under certain conditions. The participation of these bacteria in useful activities has been associated with their enzymatic activity and their establishment in specific niches. Such favorable terms may be strongly influenced by the type of fertilization supplied. Some by-products of the sugarcane industry have been produced in high amounts, and attempts have been made to continuously use them for cane production, as a way to improve soil fertility, decrease the need for chemical fertilization, and avoid environmental pollution [54]. Transcriptome sequencing and analysis of sugarcane root in PGPR strain reported in this study and it may be aptly concluded that this would be a strong initiative for the betterment of genetic studies, particularly in unraveling the functional activity and ecological behavior of PGPR in the sugarcane rhizosphere and sugarcane genomes in whole.

This study has generated the transcriptome dataset of sugarcane root in response to the plant growth-promoting rhizobacteria *Burkholderia anthina* MYSP113. A *de novo* transcriptome assembly has generated 540736 transcripts obtained from sugarcane RNA libraries. This study also identified 370 differentially expressed transcripts; among them, 199 were upregulated and 171 transcripts were down-regulated. Analysis of the enriched functional revealed that the GO terms single organism metabolic process in the molecular function category was the highly enriched one in the up-regulated group. It was also identified that the most GO terms in molecular function category to down-regulated groups were involved with the processes of transcription and translation of proteins, suggesting that the majority of the transcriptional mechanisms in sugarcane have been altered. KEGG enrichment analysis identified 238 metabolic pathways, where most of the genes were associated with carbohydrate metabolism and genetic information processing (translation & folding, sorting and degradation). KEGG enrichment analysis also showed that the metabolic pathways involved with amino acid metabolism, carbohydrate metabolism and lipid metabolism were significantly regulated, suggesting possible participation in the inherent metabolism in sugarcane root.

## Conclusion

Present work is mainly focused on comparative analysis of sugarcane root transcriptome in response to the strain MYSP113 and results demonstrate that several metabolic pathways involved in the metabolism of carbohydrates were regulated in the sugarcane root, suggesting a possible role in promoting plant growth. Moreover, further study also needed to explore the detailed pathways and genes involved in the sugarcane growth promotion and stress regulation. The results of the study contribute significantly to a better understanding of the molecular, biological and cellular mechanisms triggered in sugarcane root by *Burkholderia anthina* MYSP113. Lastly, the identification of a large number of differentially regulated transcripts opens the opportunity for the development of molecular markers associated with plant growth.

## Supporting information

S1 Fig Gene Ontology (Go) differentially expressed gene enrichment terms at 15 days (A) IT15vsCT15 down regulated DEG_Enriched_GO_classification.bar_graph (B) IT15vsCT15 up regulated DEG_Enriched_GO_classification.bar_graph.
(DOCX)

S2 Fig Gene Ontology (Go) differentially expressed gene enrichment terms at 30 days (A) IT30vsCT30 down regulated DEG_Enriched_GO_classificationbar_graph (B) IT30vsCT30 up regulated DEG_Enriched_GO_classificationbar_graph.
(DOCX)

S3 Fig Gene Ontology (Go) of differentially expressed gene enrichment terms at 45 days (A) IT45vsCT45 down regulated DEG_Enriched_GO_classificationbar_graph (B) IT15vsCT15 up ragulatedDEG_Enriched_GO_classificationbar_graph.
(DOCX)

S4 Fig KEGG_pathway_scatterplot of differentially expressed gene (DEG) enrichment at 15 days (A) IT15vsCT15_down regulated DEG_enriched_KEGG_pathway_scatterplot (B) IT15vsCT15_up regulated DEG_enriched_KEGG_pathway_scatterplot.
(DOCX)

S5 Fig KEGG_pathway_scatterplot of differentially expressed gene (DEG) enrichment at 35 days (A) IT30vsCT30_ down regulated DEG_enriched_KEGG_pathway_scatterplot (B) IT30vsCT30_up regulated DEG_enriched_KEGG_pathway_scatterplot.
(DOCX)

S6 Fig KEGG_pathway_scatterplot of differentially expressed gene (DEG) enrichment at 45 days (A) IT45vsCT45_down regulated DEG_enriched_KEGG_pathway_scatterplot (B) IT45vsCT45_up regulated DEG_enriched_KEGG_pathway_scatterplot.
(DOCX)

S7 Fig Validation of DEGs obtained from RNA-seq using qRT-PCR. The blue and red bars represent the fold change of the relative expression level from the RNA-seq and qRT-PCR data, respectively. The sugarcane GAPDH gene, as an internal control, was used to normalize the expression levels of the target genes. Bars represent the standard error of the mean (n = 3).
(DOCX)

S1 Table Gene-specific primers used in gene expression analysis by qRT-PCR. (DOCX)

S2 Table The ratio of successfully annotated Genes. (DOCX)
