# Supplementary figures and images for: Comparative analysis of sugarcane root transcriptome in response to the plant growth-promoting Burkholderia anthina MYSP113
Source: PLoS One. 2020 Apr 8;15(4):e0231206. doi: 10.1371/journal.pone.0231206 (PMC7141665; doi:10.1371/journal.pone.0231206)

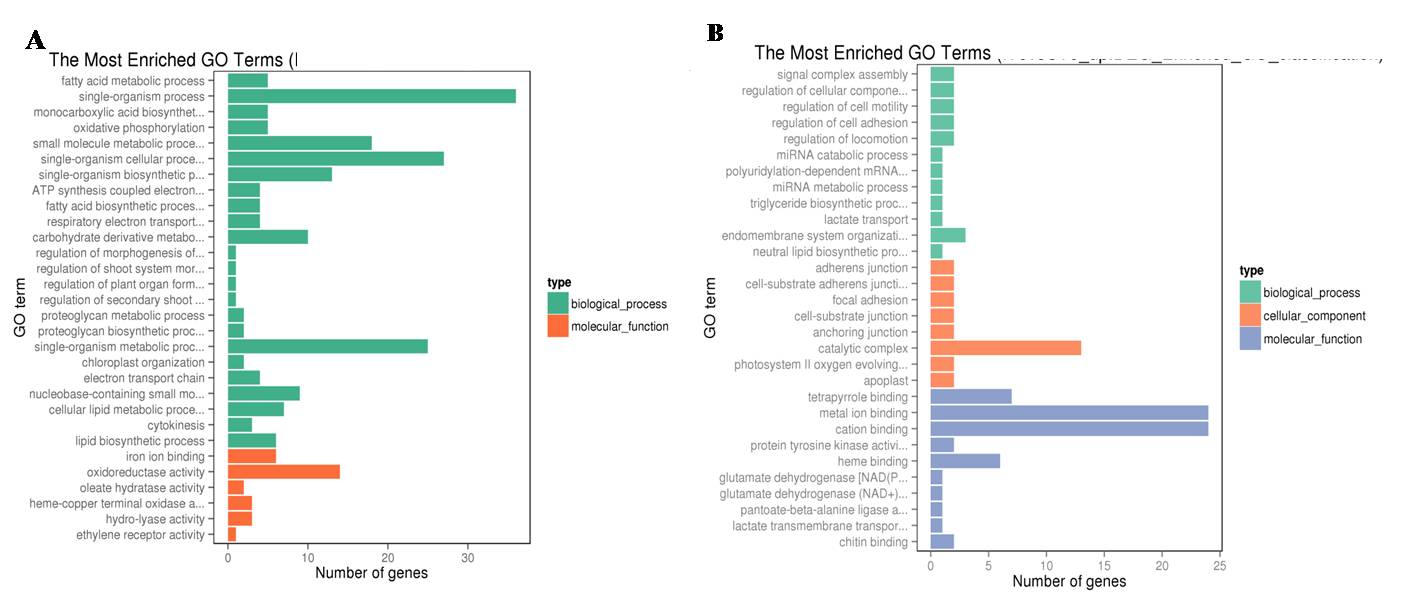


**Fig. S1**

Supplement: S1 Fig — Gene Ontology (Go) differentially expressed gene enrichment terms at 15 days (A) IT15vsCT15 down regulated DEG_Enriched_GO_classification.bar_graph (B) IT15vsCT15 up regulated DEG_Enriched_GO_classification.bar_graph. (DOCX) [file pone.0231206.s001.docx]

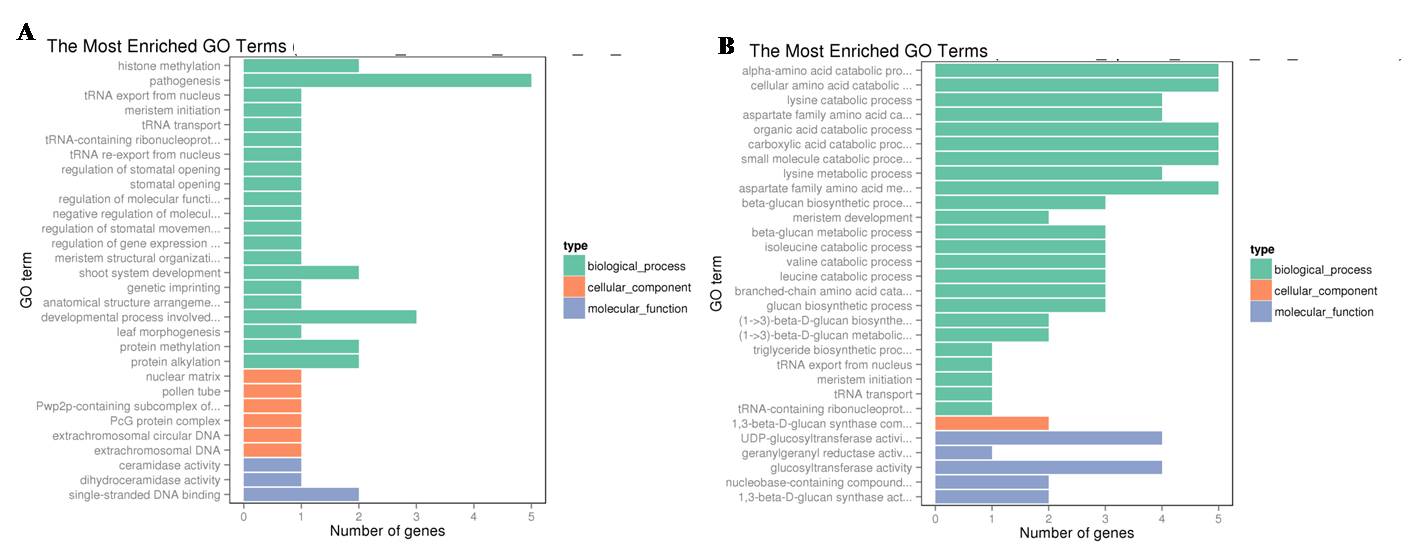


**Fig. S2**

Supplement: S2 Fig — Gene Ontology (Go) differentially expressed gene enrichment terms at 30 days (A) IT30vsCT30 down regulated DEG_Enriched_GO_classificationbar_graph (B) IT30vsCT30 up regulated DEG_Enriched_GO_classificationbar_graph. (DOCX) [file pone.0231206.s002.docx]

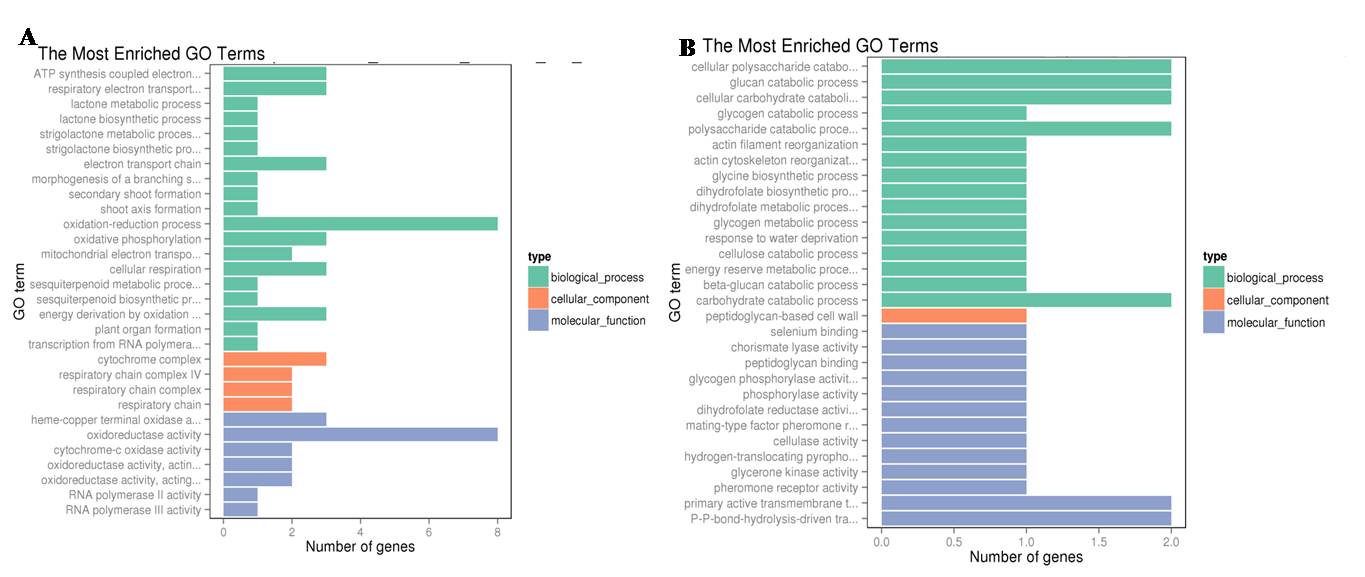


**Fig. S3**

Supplement: S3 Fig — Gene Ontology (Go) of differentially expressed gene enrichment terms at 45 days (A) IT45vsCT45 down regulated DEG_Enriched_GO_classificationbar_graph (B) IT15vsCT15 up ragulatedDEG_Enriched_GO_classificationbar_graph. (DOCX) [file pone.0231206.s003.docx]

**
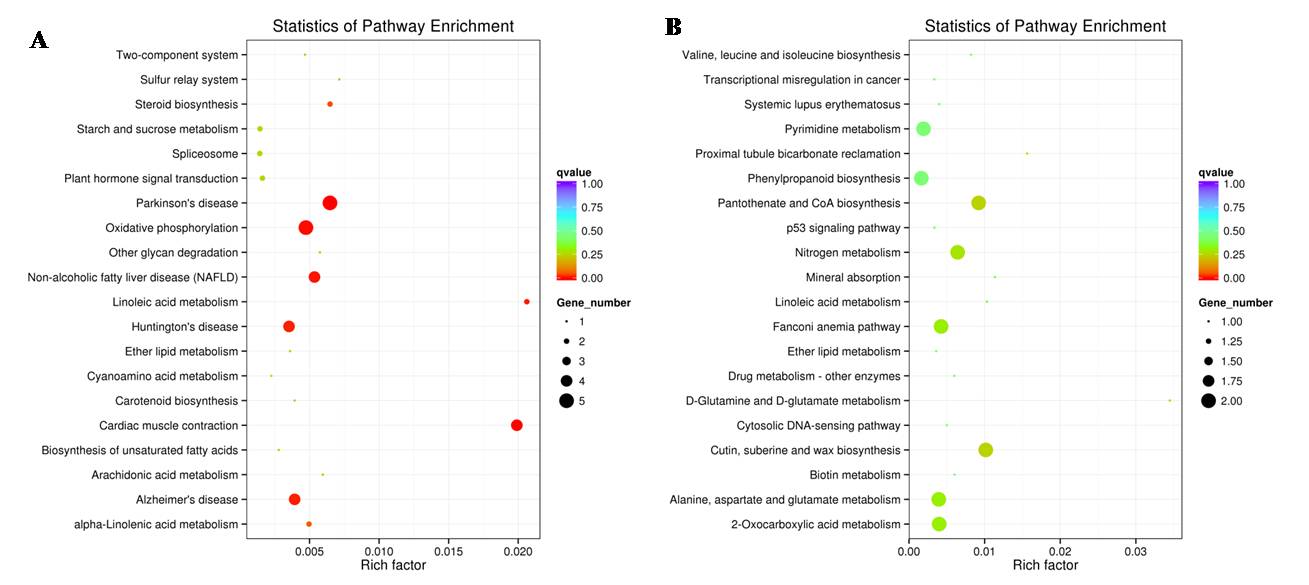
**

**Fig. S4**

-

Supplement: S4 Fig — KEGG_pathway_scatterplot of differentially expressed gene (DEG) enrichment at 15 days (A) IT15vsCT15_down regulated DEG_enriched_KEGG_pathway_scatterplot (B) IT15vsCT15_up regulated DEG_enriched_KEGG_pathway_scatterplot. (DOCX) [file pone.0231206.s004.docx]

**
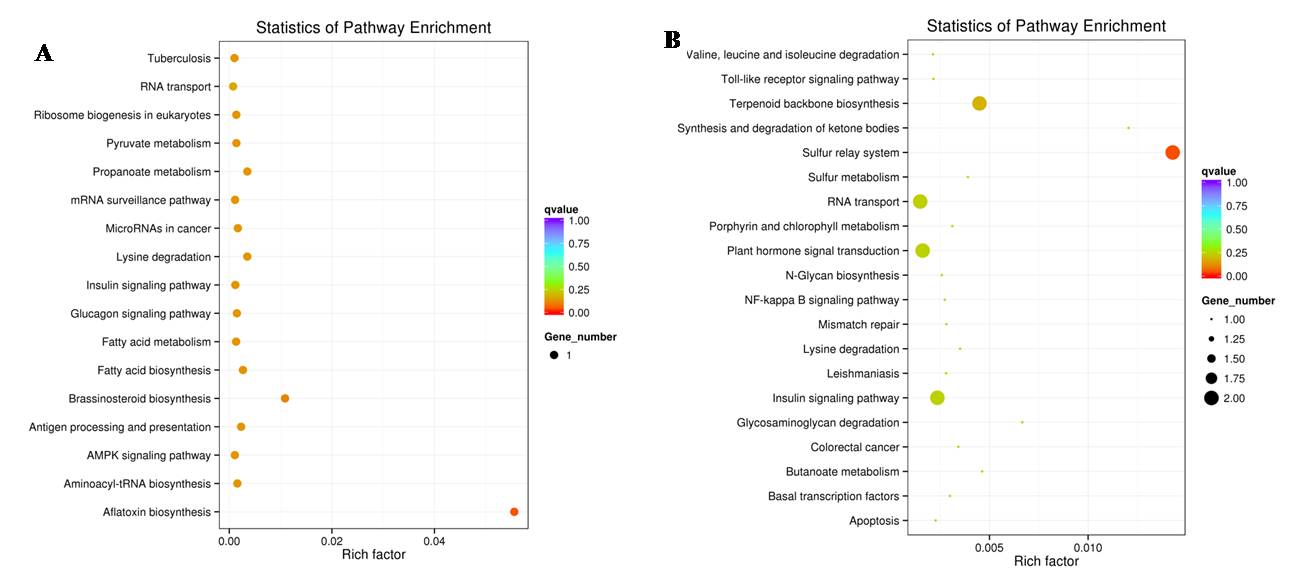
**

**Fig. S5**

Supplement: S5 Fig — KEGG_pathway_scatterplot of differentially expressed gene (DEG) enrichment at 35 days (A) IT30vsCT30_ down regulated DEG_enriched_KEGG_pathway_scatterplot (B) IT30vsCT30_up regulated DEG_enriched_KEGG_pathway_scatterplot. (DOCX) [file pone.0231206.s005.docx]

**
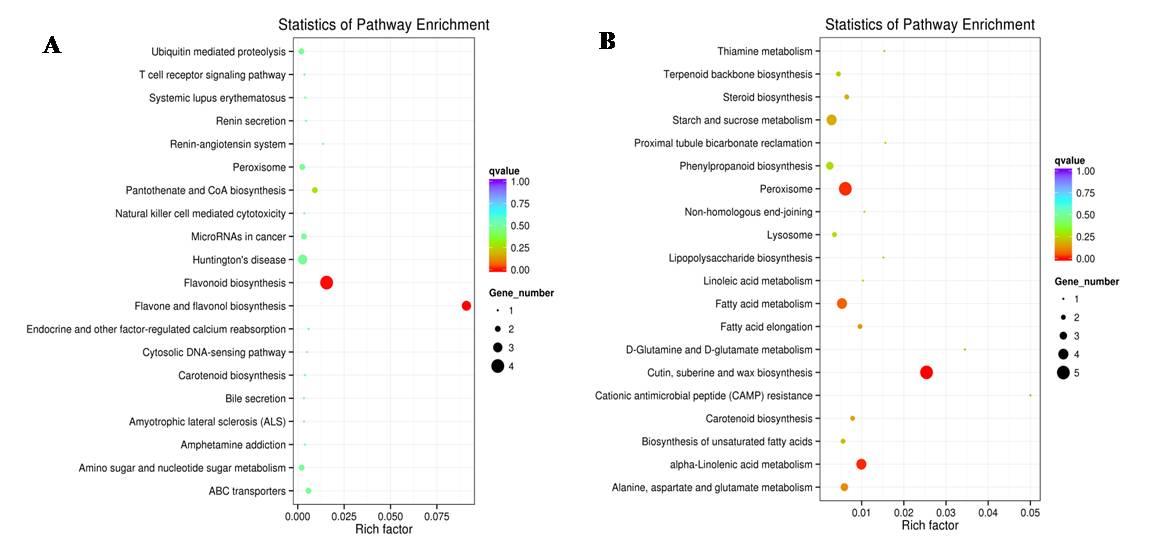
**

**Fig. S6**

Supplement: S6 Fig — KEGG_pathway_scatterplot of differentially expressed gene (DEG) enrichment at 45 days (A) IT45vsCT45_down regulated DEG_enriched_KEGG_pathway_scatterplot (B) IT45vsCT45_up regulated DEG_enriched_KEGG_pathway_scatterplot. (DOCX) [file pone.0231206.s006.docx]

**
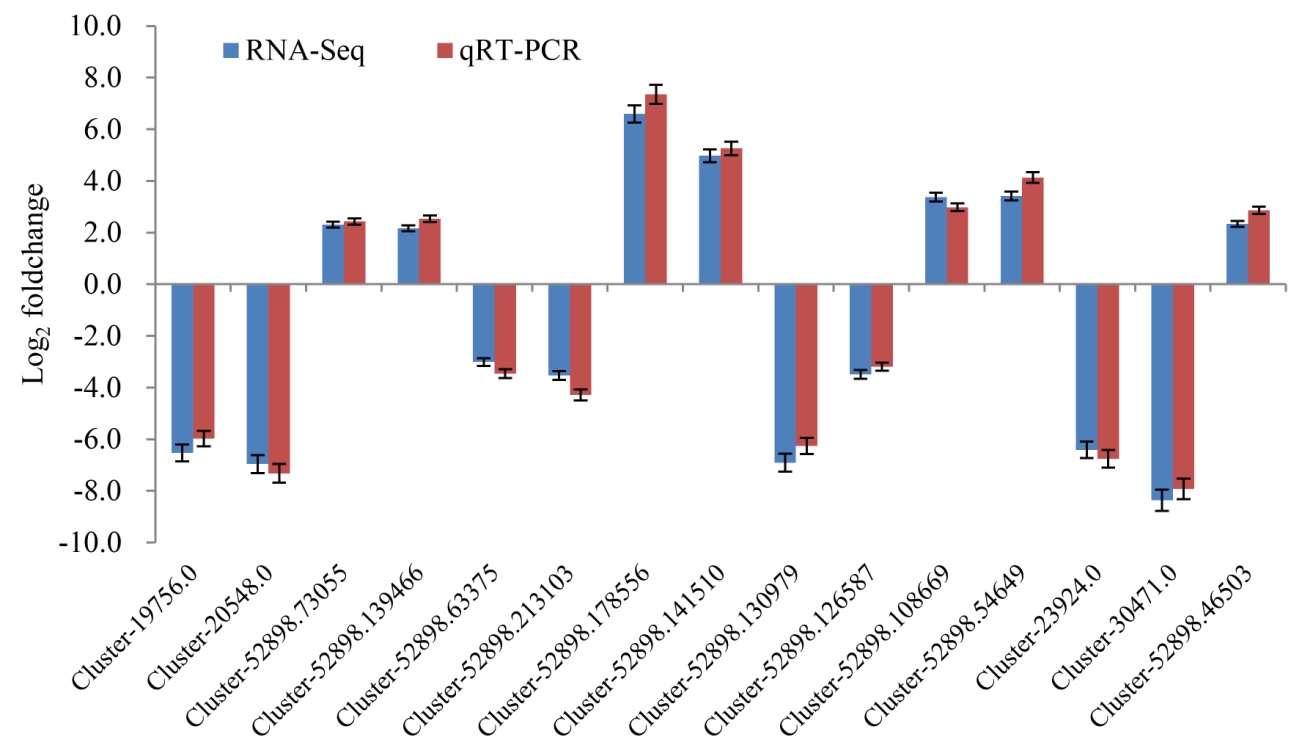
**

**Fig. S7**

Supplement: S7 Fig — The blue and red bars represent the fold change of the relative expression level from the RNA-seq and qRT-PCR data, respectively. The sugarcane GAPDH gene, as an internal control, was used to normalize the expression levels of the target genes. Bars represent the standard error of the mean (n = 3). (DOCX) [file pone.0231206.s007.docx]
