# Supplementary material for: Comparative analysis of sugarcane root transcriptome in response to the plant growth-promoting Burkholderia anthina MYSP113
Source: PLoS One. 2020 Apr 8;15(4):e0231206. doi: 10.1371/journal.pone.0231206 (PMC7141665; doi:10.1371/journal.pone.0231206)
Supplement: S1 Table — (DOCX) [file pone.0231206.s008.docx]

**Table S1. Gene-specific primers used in gene expression analysis by qRT-PCR**

| Gene ID | Description | Primer |
| --- | --- | --- |
| EF189713 | Glyceraldehyde-3-phosphate dehydrogenase (GAPDH) | F: TGGTGCTGACTATGTCGTGGA |
|  |  | R: CATGGGTGCATCTTTGCTTG |
| Cluster-23924.0 | Steroid 17-alpha-hydroxylase | F: ACAACACTACCTCAGACCGG |
|  |  | R: CGGCCGAAACCTACATTCTG |
| Cluster-30471.0 | Cytochrome c oxidase | F:AATACCCGTTAAGCCACCCA |
|  |  | R: AGGCCTAGACGTAGACACAC |
| Cluster-52898.46503 | Non-symbiotic hemoglobin | F: AGCTTGTCATCCGTTCATGG |
|  |  | R: CGCAGGAAGGAGAACATCTG |
| Cluster-19756.0 | NADH dehydrogenase | F: CGAGTTGGAATTGGCTAGGC |
|  |  | R: AATCGCCTCTCCTCCGTAAG |
| Cluster-20548.0 | Cytochrome c oxidase | F: TGGCCGAATATAGTCCTGGTC |
|  |  | R: ACACTCATGAGCTGTTCCATC |
| Cluster-52898.73055 | Calcium binding EF-hand protein | F: TCTTTGACGAGGACAGGGAC |
|  |  | R: CAAAGGACCGAAAGCAGCAT |
| Cluster-52898.139466 | Peroxidase 2 | F: CAAATAAGCTCGCCGTCCTC |
|  |  | R: GGGCTTGGTGGAGGATAAGT |
| Cluster-52898.63375 | NAC domain-containing protein | F: TTTGGCTGACATGGTTGAGC |
|  |  | R: AGAACCAACTTTTCCACGGC |
| Cluster-52898.213103 | Pentatricopeptide repeat-containing protein | F: ACTGATCCCTTCCATGGCAA |
|  |  | R: CAAGGCCTGAACTGACCAAC |
| Cluster-52898.178556 | Aldehyde dehydrogenase | F: CTCCACTTCCAGTACAGCCA |
|  |  | R: CACCTTGTTCACAACCTCCG |
| Cluster-52898.141510 | Dof zinc finger protein | F: AGGTTGCTCTTCCCCTTCG |
|  |  | R: TGCCATTCCCGATCAAGCTA |
| Cluster-52898.130979 | Glucuronosyltransferase | F: TGGTGGAGTTGTTGCTTTCG |
|  |  | R: TCCCTTGAAAACCGCACATG |
| Cluster-52898.126587 | Microtubule-associated protein | F: GATGTTTGGGAAGCTCGGTC |
|  |  | R: CCTGTGCCAAACTTCCCATG |
| Cluster-52898.108669 | Ribonuclease H protein | F: AATGGGTCGTGTGGTGGTAT |
|  |  | R: GGAGAGGAAGGAGCGTAGAC |
| Cluster-52898.54649 | Chorismatelyase | F: TGAGGCTACGATCTGCACAT |
|  |  | R: TGCAGATGGACTCGGATCTC |

Note: Primers of F/R represent forward/reverse primers.
