# Supplementary material for: Comparative analysis of sugarcane root transcriptome in response to the plant growth-promoting Burkholderia anthina MYSP113
Source: PLoS One. 2020 Apr 8;15(4):e0231206. doi: 10.1371/journal.pone.0231206 (PMC7141665; doi:10.1371/journal.pone.0231206)
Supplement: S2 Table — (DOCX) [file pone.0231206.s009.docx]

**Table S2. The ratio of successfully annotated genes**

| Annotations | Number of Unigenes | Percentage (%) |
| --- | --- | --- |
| Annotated in NR | 89765 | 28.49 |
| Annotated in NT | 190153 | 60.37 |
| Annotated in KO | 63741 | 20.23 |
| Annotated in SwissProt | 126321 | 40.1 |
| Annotated in PFAM | 136351 | 43.29 |
| Annotated in GO | 148930 | 47.28 |
| Annotated in KOG | 47988 | 15.23 |
| Annotated in all Databases | 11699 | 3.71 |
| Annotated in at least one Database | 232348 | 73.76 |
| Total Unigenes | 314967 | 100 |
